# Frailty and COVID-19: A Systematic Scoping Review

**DOI:** 10.3390/jcm9072106

**Published:** 2020-07-04

**Authors:** Giuseppe Maltese, Andrea Corsonello, Mirko Di Rosa, Luca Soraci, Cristiana Vitale, Francesco Corica, Fabrizia Lattanzio

**Affiliations:** 1 Department of Diabetes and Endocrinology, Epsom & St Helier University Hospitals, Surrey SM5 1AA, UK; giuseppe.maltese@kcl.ac.uk; 2 Unit for Metabolic Medicine, Cardiovascular Division, Faculty of Life Sciences & Medicine, King’s College, London WC2R 2LS, UK; 3 Unit of Geriatric Pharmacoepidemiology and Biostatistics and Unit of Geriatric Medicine, IRCCS INRCA, 60124 Ancona, Italy; m.dirosa@inrca.it (M.D.R.); drlucasoraci89@gmail.com (L.S.); 4 Department of Clinical and Experimental Medicine, University of Messina, 98124 Messina, Italy; coricaf@unime.it; 5 Department of Medical Science, IRCCS San Raffaele Pisana, 00163 Rome, Italy; cristiana.vitale@gmail.com; 6 Scientific Direction, IRCCS INRCA, 60124 Ancona, Italy; f.lattanzio@inrca.it

**Keywords:** coronavirus disease-19 (COVID-19), frailty, mortality, older

## Abstract

Older people have paid a huge toll in terms of mortality during the coronavirus disease-19 (COVID-19) pandemic. Frailty may have contributed to the vulnerability of older people to more severe clinical presentation. We aimed at reviewing available evidence about frailty and COVID-19. We searched PUBMED, Web of Science, and EMBASE from 1 December 2019 to 29 May 2020. Study selection and data extraction were performed by three independent reviewers. Qualitative synthesis was conducted and quantitative data extracted when available. Forty papers were included: 13 editorials, 15 recommendations/guidelines, 3 reviews, 1 clinical trial, 6 observational studies, 2 case reports. Editorials and reviews underlined the potential clinical relevance of assessing frailty among older patients with COVID-19. However, frailty was only investigated in regards to its association with overall mortality, hospital contagion, intensive care unit admission rates, and disease phenotypes in the few observational studies retrieved. Specific interventions in relation to frailty or its impact on COVID-19 treatments have not been evaluated yet. Even with such limited evidence, clinical recommendations on the use of frailty tools have been proposed to support decision making about escalation plan. Ongoing initiatives are expected to improve knowledge of COVID-19 interaction with frailty and to promote patient-centered approaches.

## 1. Introduction

The first cases of coronavirus disease-19 (COVID-19), caused by a novel coronavirus SARS-CoV-2, were first identified in Wuhan, China. The disease spread rapidly and reached the criteria to be regarded as a pandemic in March 2020 [1]. As of 30 May 2020, COVID-19 has infected nearly 6 million people around the world and caused more than 350,000 deaths.

Initial reports from Wuhan revealed that most cases of COVID-19 have occurred in people aged 60 or above, and fatality rates rose exponentially with age, from 0.4% among those aged 40–49 years to 3.6% among those aged 60–69 years and 14.8% among those aged >80 years [2]. Additional reports from Italy and the UK, which are among the most hit countries in Europe, have confirmed the high risk of death in older adults, particularly in those with pre-existing diseases (e.g., cardiovascular and respiratory diseases, obesity, diabetes, chronic kidney disease, cancer, gastrointestinal, skin, muscle-skeletal, and immune diseases) [3,4,5]. Data from the Italian Institute of Health (ISS) report of 28 May 2020 showed that the mean age of patients dying for COVID-19 was 80 years (median 82, range 0–100, IQR 74–88). Italian data, however, do not entirely account for deaths in care homes. Therefore, it is likely that the mean age of patients dying for COVID-19 could be higher. Among patients who died, 3.5% were aged 50–59 years, 10.3% were aged 60–69 years, 26.9% were aged 70–79 years, 40.9% were aged 80–89 years, and 17.2% were aged 90 or more. Finally, people who died had at least one comorbid condition in 95.9% of cases [6]. Similarly, a large-scale study conducted in the UK, including 20,133 hospitalized patients, revealed that the median age of patients admitted to hospital with COVID-19 or with a diagnosis made in hospital, was 73 years. The median age of patients who died in hospital from COVID-19 in the study was 80 years, and only 11% of these patients had no documented major comorbidity [4].

Besides revealing the unpreparedness of some Healthcare Systems worldwide (e.g., Italy and Spain), with the hospitals overstretched due to the increasing number of patients admitted, the lack of intensive care unit (ICU) beds and thousands of older people dying at home, in hospitals, and in nursing facilities, COVID-19 is currently prompting research on reasons underlying such increased vulnerability of older patients to the most negative consequences of the infection.

Frailty is a condition characterized by declining function across several homeostatic systems leading to increased vulnerability to stressors and risk of adverse health outcomes [7,8]. Thus, it is very likely that frailty, together with comorbidities, may have contributed to the high vulnerability to severe clinical manifestations and deaths from COVID-19 among older people. During the pandemic, a systematic use of frailty assessment in all elderly patients could have identified frail older people in the early stage of COVID-19. This could have allowed a better allocation of care resources to those at higher risk of unfavorable outcome and could also have avoided excessive hospital admissions which turned out to be amplifiers of the COVID-19 pandemic worldwide. Therefore, the aim of this systematic scoping review was to summarize the available evidence on frailty assessment during the COVID-19 pandemic, in an attempt to identify knowledge gaps to be bridged and possibly to draw future perspectives in this field.

## 2. Methods

This scoping review is reported in accordance with the Extended Preferred Reporting Items for Systematic Reviews and Meta-Analyses Statement for Scoping Reviews (PRISMA-ScR) [9].

### 2.1. Data Sources and Searching

We conducted a systematic literature review on Pubmed, Embase, and Web of Science using the following search strategies.

Pubmed and Web of Science:

(Coronavirus* OR “Coronavirus Infection” OR “COVID-19” OR “Coronavirus Infection Disease 2019” OR “2019 Novel Coronavirus Infection” OR “2019-nCoV Infection” OR “2019 nCoV Infection” OR “2019-nCoV Infections” OR “SARS-CoV-2” OR “Novel Coronavirus Pneumonia” OR “2019 novel coronavirus” OR “coronavirus disease 2019” OR “nCoV” OR COVID*) AND Frail*

Embase:

Coronaviruses OR ‘coronavirus infection’/exp OR ‘coronavirus infection’ OR ‘COVID-19’ OR ‘coronavirus infection disease 2019’ OR ‘2019 novel coronavirus infection’ OR ‘2019-ncov infection’ OR ‘2019 ncov infection’ OR ‘2019-ncov infections’ OR ‘SARS-CoV-2’ OR ‘novel coronavirus pneumonia’ OR ‘2019 novel coronavirus’/exp OR ‘2019 novel coronavirus’ OR ‘coronavirus disease 2019’ OR ‘ncov’ OR COVID*

AND frail*

The time frame of the literature search ranged between December 1, 2019 and May 29, 2020. Only English language studies were selected for evaluation. A manual search of reference lists of relevant papers was performed to identify additional articles.

### 2.2. Eligibility Criteria

Three assessors (MDR, LC, AC) independently screened title and abstract of the records retrieved from the medical literature. The following eligibility criteria were used to retrieve studies to be included in the review:

- Type of publication: all types of publications were considered, including editorials, recommendations/guidelines, cross-sectional or cohort (retrospective and prospective) studies, and clinical trials.
- Participants: only studies including people older than 65 years were included for evaluation.
- Comparators: studies comparing different methods to assess frailty were searched. However, in order to obtain a comprehensive scoping review, papers investigating only one frailty assessment method were included.
- Outcomes: retrieved papers were assessed independently of the study outcome. Data on mortality and/or functional outcomes were extracted whenever available.

The full text of each article selected by at least one of the assessors was fully evaluated. The same assessors extracted independently information from the selected papers. Any disagreement was resolved through consensus building in the focus group. Data were grouped according to the type of publication.

## 3. Results

**Figure 1:**
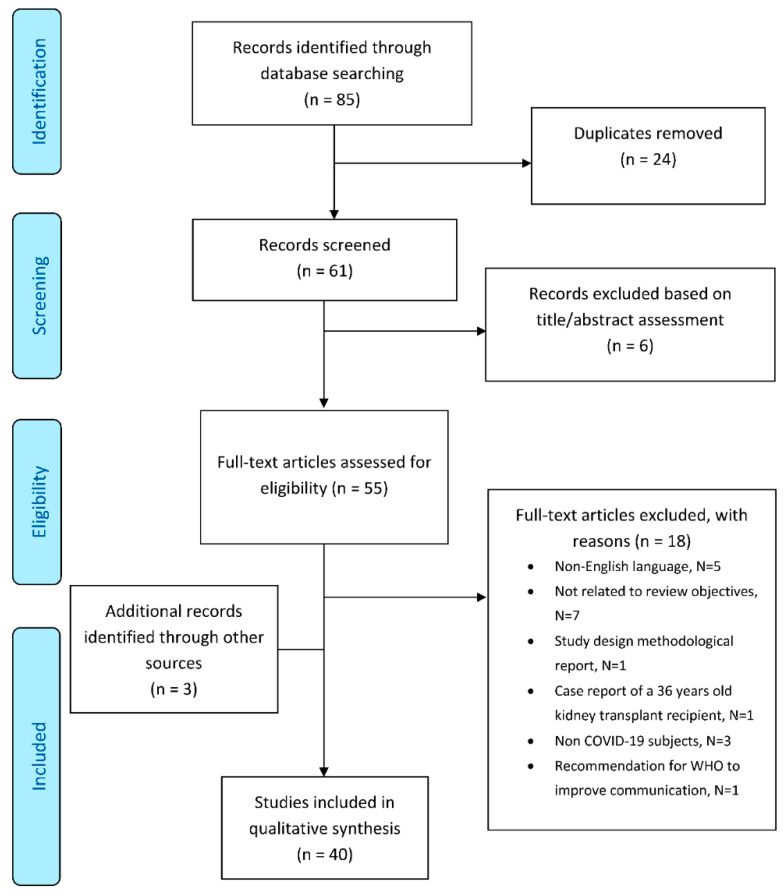
Scoping literature review flow-chart (PRISMA).

Figure 1 shows information on literature review methodology and the reasons for inclusion and exclusion of identified citations. The electronic search strategy identified a total number of 85 citations. Of these, 61 were considered potentially eligible on the basis of title/abstract evaluation and included in full-text assessment. Fifty-five publications were initially selected, and 18 were excluded during full-text assessment. Three publications were retrieved after careful assessment of the reference lists of publications included in the review, leading to a final number of 40 publication to be examined: 13 editorials, 15 recommendations/guidelines, 3 reviews, 1 clinical trial, 6 observational studies, 2 case reports.

### 3.1. Overview of Included Editorials

Overall, 13 editorials were retrieved and assessed. The need of assessing frailty among older patients with COVID-19 clearly emerged in all the editorials [10,11,12,13,14,15,16,17,18,19,20,21,22].

Among the 13 editorials retrieved, seven underlined the importance of a geriatric approach and suggested to profile risks using a comprehensive geriatric assessment [10,11,12,13,14,16,22]. The need for an ad-hoc, easy-to-administer frailty assessment tool to evaluate the health status of older patients with COVID-19 was proposed in seven editorials [10,13,14,15,17,18,19]. Five editorials highlighted that such ad-hoc frailty assessment could be more determinant than age in the assessment of the risk of poor health outcomes or ICU admission, avoiding ageism in a framework of limited resources [12,13,16,18,19,21,22]. Frailty assessment was also considered relevant to ensure the development of vaccines with a favorable immune response in frail individuals [19]. Nevertheless, Hubbard et al. acknowledged limitations of frailty assessments with the Clinical Frailty Scale (CFS) when used in the hospital setting or to determine access of older people to health care [15]. Indeed, this tool, being a screening test, may help to predict poor health outcomes but is not intended to identify end-of-life patients and should not be used to decide on the allocation of healthcare resources. Additionally, relevant cut-offs for the access of older people to health care have yet to be investigated [15].

Isolation was another important issue raised by a few editorials: two editorials warned about the negative outcomes of social isolation for older frail people [14,22], two stressed the need of remote monitoring systems specifically devoted to isolated patients [10,16], and three highlighted the negative effects of isolation in nursing homes [10,20,22]. Finally, two editorials reminded of the importance of an appropriate management of chronic conditions among older frail patients during a pandemic [10,14], and two stressed the strategic importance of testing vulnerable populations to face the spread of the infection [10,22].

### 3.2. Overview of Included Recommendations/Guidelines

Among the 15 recommendations/guidelines retrieved, seven underlined the need for frailty assessment of hospitalized subjects with COVID-19, almost exclusively to support decision making for ICU allocation or ventilation [23,24,25,26,27,28,29]. Clarfield et al. emphasized the role of active palliative care at the end of life during COVID-19 and recommended that very frail older people with severe dementia should be actively protected from ventilator-associated complications and subsequent risk of undignified death [28]. Three recommendations, specifically developed for people shielding at home, highlighted the importance of physical exercise in preventing loss of muscle strength, flexibility, and aerobic capacity [30,31,32]. Four guidelines were focused on the management of patients with specific diseases, including diabetes, hemophilia, and cancer [33,34,35,36], and one focused on palliative care for older frail patients [37]. In terms of the setting, eight guidelines were specific for hospitals or nursing homes [23,24,25,26,27,28,29,34] and six for home care [30,31,32,33,35,36], while one recommendation was made for both settings [37]. With regards to the frailty index, four guidelines suggested to screen patients with the Clinical Frailty Scale [23,27,29,34], and one suggested the French Clinical Frailty Score or Frailty Group Iso-Resource Evaluation (FRAGIRE) score [26], while all the others did not specify any frailty index.

### 3.3. Overview of Included Reviews

Of the three reviews retrieved, two underlined the importance of frailty assessment in the management of COVID-19 sequelae among affected subjects [38,39]. The third review proposed the implementation of frailty assessment and prehabilitation for pre-operative patients who had their surgeries delayed or will have newly scheduled procedures during the peri-pandemic period [40]. Frailty was considered a marker of increased morbidity and mortality burden [38,39], but also a potential consequence of COVID-19 pandemics-related isolation measures in both affected and nonaffected individuals [40]. Silver et al. warned about the impact of social distancing measures and isolation on individuals’ physical and mental health, including decreased physical activity with subsequent cardiac deconditioning, increased tobacco use, and poor nutrition and glycemic control, which may lead to poor surgical outcomes [40]. All reviews suggested the adoption of preventive measures, such as regular physical exercise and the use of functional food [38,39,40], smoking cessation [38,40], tight glycemic control in diabetic patients [40], and psychological support [38]. Finally, the potential use of several anti-aging compounds able to contrast immunosenescence was proposed and warrants further investigations [38,39].

### 3.4. Overview of Included Clinical Trials

The only open randomized trial included in the present review investigated the effects of a six-week respiratory rehabilitation training in elderly patients with COVID-19 (intervention group *N* = 36, control group *N* = 36). Although frailty was not formally assessed in this study, the functional status before entering the study was evaluated by the Functional Independence Measure (FIM). Additionally, frailty-related outcomes, including respiratory function, quality of life, mobility, and psychological function, were assessed in this study. Interventions included respiratory muscle training, cough exercise, diaphragmatic training, stretching exercise, and home exercise. Six-week respiratory rehabilitation was found to improve respiratory function, quality of life, and anxiety in elderly patients with COVID-19, but it had no significant impact on mood and activities of daily living [41].

### 3.5. Overview of Included Observational Studies

Five observational studies included in this review investigated the predictive role of frailty among COVID-19 patients admitted to hospitals [42,43,44,45,46]. Baker et al. simply reported that patients who died without ventilatory support were generally frail with a median Clinical Frailty Scale score of 7 (interquartile range = 6–7) [47] (Table 1). The Clinical Frailty Scale (CFS) was used for frailty assessment in five studies [43,44,45,46,47]. Only one study used the 31-item Frailty Index [42]. The primary outcomes were overall mortality [42,43,44,45,46,47], hospital infection [43], ICU admission rates [42], and disease phenotypes [45]. Interestingly, studies using the CFS led to heterogeneous results. A high CFS score was associated with an increased hospital mortality in two studies [43,46] and with a higher risk of nosocomial infection in one study [43]. Miles et al. reported that frailty may be associated with an increased mortality in non-COVID-19 patients as compared to those tested positive for COVID-19 [44]. High CFS was found associated with longer disease course and prolonged dying phases [45]. Older age and a 31-item Frailty Index qualified as independent predictors of hospital mortality or ICU admission [42].

### 3.6. Overview of Included Case Reports

Two papers describing atypical presentations of COVID-19 were reviewed. The first case report compared two older patients. An 83-year-old patient presented to the emergency department after a fall. He had no history of fever or cough. Because of a thoracic trauma, the patient underwent a chest computed tomography that incidentally revealed subpleural, ground-glass opacities with the air bronchogram sign. A nasal-pharyngeal swab was positive for SARS-Cov-2, confirming the diagnosis of COVID-19 and leading to an immediate admission. The second patient, an 80-year-old woman living in a nursing home where two other residents were already diagnosed with COVID-19, presented to the emergency department with dyspnea and cough. Nasal-pharyngeal swabs resulted negative for SARS-CoV-2. After an extensive work-up, decompensated heart failure was deemed the most likely cause of diffuse lung infiltrates on the chest X-ray [48].

The authors of the second paper reported on a 94-year-old man with well-controlled schizoaffective disorder, who presented with delirium, low-grade pyrexia, and abdominal pain. He was initially given antibiotics for an infection of unknown origin, and subsequently switched to treatment for community-acquired pneumonia. Despite active treatment, he became tachypneic with deteriorating oxygen saturation. A repeat chest X-ray showed widespread opacification. A postmortem throat swab identified was consistent with COVID-19 infection [49].

## 4. Discussion

During the COVID-19 pandemic, the need for a careful frailty evaluation only partly emerged as a clinically relevant tool to assess vulnerable older people. Despite several editorials [10,11,12,13,14,15,16,17,18,19,20,21,22] and reviews [38,39,40] underlining the potential relevance of assessing frailty among older people affected by COVID-19, only a few observational studies were carried out [42,43,44,45,46,47], resulting in a very limited production of new clinical data to be rapidly implemented into the care process. Indeed, frailty was only investigated in regards to its association with overall mortality [42,43,44,45,46,47], hospital infection [43], ICU admission rates [42], and disease phenotypes [45] in the available studies. Specific interventions in relation to frailty or its impact on COVID-19 treatments were not evaluated. Nevertheless, even in the context of such limited evidence, clinical recommendations on the use of frailty tools were proposed, mainly to support decision making about an escalation plan and to avoid ageism [23,25,28]. Furthermore, during the COVID-19 pandemic the different strategies adopted in different countries with selective admission of younger patients and do-not-resuscitate labelling have highlighted the problem of ageism. It is worth noting that frailty should never be considered as a synonym for end of life when deciding about allocation of limited health care resources [15]: several studies showed that most frail patients do survive acute illness and return home after hospitalization [50], and currently, there are no sufficient data to affirm that this is not true in the context of COVID-19 pandemic. Additionally, appropriate cut-off points for the use of frailty scales to determine access of older people to health care have yet to be investigated. Nevertheless, CFS is an easy-to-use and reliable screening tool to identify frailty, which was validated also in the emergency department where it may provide very useful clinical information [51]. Thus, a patient-centered approach to older COVID-19 patients should always consider the clinical severity of illness presentation and likelihood of success of interventions together with the degree of frailty [15].

The frailty conceptual framework describes a condition of increased vulnerability to stressors due to declining function of homeostatic mechanisms and consequent increased risk of adverse health outcomes [7,8]. Thus, it is very likely that frailty, together with comorbidities, may contribute to the high vulnerability and increased risk of death of older people with COVID-19 infection. Frailty definition has mainly a preventive meaning, since assessing frailty may help physicians to identify those older people who need close monitoring of their functional status and specific interventions aimed at reducing the risk of negative outcomes. The task force of the International Conference of Frailty and Sarcopenia Research (ICFSR) recommended that health practitioners screen all older adults for frailty by using a validated setting-specific instrument. Recommendations also suggest that primary care may represent the most appropriate setting to identify older adults with frailty and try to prevent adverse outcomes through individualized plans. For subjects screened positive for frailty, a more comprehensive assessment should be performed to identify signs and underlying mechanisms of frailty (strong recommendations) [52]. There is a strong rationale for using frailty screening information in the context of COVID-19 pandemic. Together with the estimation of illness severity and likelihood of successful intervention, frailty screening could be very useful in primary care to assign the right patient to the right setting and maybe avoid the excessive hospitalizations, which turned out to be amplifiers of the COVID-19 pandemic worldwide. Since 2017, in the UK, the General Medical Services (GMS) have instructed GPs to use the electronic Frailty Index (eFI) to identify patients aged 65 and over with moderate or severe frailty [53]. In Italy, there is a national initiative on frailty prevention and an agreed plan to sustain it [54], but there is still no widespread use of frailty screening outside geriatric wards. Indeed, the triage of patients with COVID-19 was based only on symptoms and clinical parameters according to local guidelines, and no information about the functional status was required.

A growing body of evidence indicates that COVID-19 can occur with atypical presentations, especially in the elderly. In Italy, 24% of COVID-19 patients who died during pandemic had no fever, 27% had no dyspnea, and 61% had no cough at presentation [6]. There is also description of older people with COVID-19 presenting with a history of falls or delirium [48,49], which further suggests the need for an early assessment of frailty and careful monitoring of physical and cognitive status during a period of social distancing and self-isolation.

Social distancing and self-isolation, key means to slow down the coronavirus spread, also had implications on the health of older individuals. During the pandemic, in some countries, older people had to face the challenges related to food supply and long queues to enter supermarkets. In addition, the lack of physical activity may have contributed to muscle mass loss, weakness, and falls. Again, the availability of an early assessment of frailty could have been extremely useful to identify patients at risk of deterioration due to these factors.

Interesting care models exist and should be taken into consideration in managing community-dwelling frail older people. For instance, telemedicine and wearable technology as tools to monitor the health status remotely and identify changes in frailty were proposed [22]. However, the use of technology may pose considerable challenges for some old individuals, particularly the ones with cognitive impairment, hearing loss, and other common sensory and motor deficits for which community health workers may be rapidly employed or redeployed from suspended activities to provide home care support [55].

Finally, post-COVID-19 patients may exhibit extra-pulmonary manifestations, including neurological, cardiovascular, and musculoskeletal disorders, burdening functional status [56,57]. Hence, most of the older patients may require functional, neuromotor, respiratory, and cardiac rehabilitation, which warrants frailty assessment. The only clinical trial retrieved in the present review showed that respiratory rehabilitation may significantly improve functional outcomes [41]. Additionally, the COVID-19 pandemic increased both the pressure from acute care wards to transfer patients to rehabilitation services and the difficulty in providing outpatient and home-based rehabilitation, which requires a rapid re-shaping of the organization of rehabilitation services. A recent review pointed out that an early rehabilitation should be granted to inpatients with COVID-19; exercise programs should be provided to older people during social isolation to reduce the risk of frailty, sarcopenia, cognitive decline, and depression; and telerehabilitation may represent the first rehabilitation option for people at home [31].

Several frailty-related topics have not yet been addressed and warrant further investigations. These include risk profiling based on comprehensive geriatric assessment and testing markers of frailty in relation to outcomes, cost/benefit analysis of selected COVID-19 treatments, detection of early warning symptoms and atypical presentations among patients with multimorbidity, use of remote monitoring and teleconsultation for isolated patients with chronic conditions, provision of psychological support, and definition of age- and frailty-specific recommendations for ventilatory assistance. Nursing home safety criteria need to be standardized and strictly monitored to avoid the spread of COVID-19 amongst the most frail and vulnerable population. The impact of sensorial, cognitive, and emotional consequences in COVID-19 survivors also deserve to be studied [10].

### Limitations and Future Perspectives

Limitations of this study deserve to be mentioned. The intention of a scoping review is simply to summarize the breadth of the available literature, and it refrains from assessing the quality of studies. Several editorials, recommendations/guidelines, and reviews were included in the present study, and most of the studies reporting data on frailty in COVID-19 patients had important limitations, including the use of small population samples and simple comparisons. Additionally, papers in languages other than English were excluded.

Nevertheless, our study underlines the need for a rapid response from the geriatric scientific community to the COVID-19 challenge [22]. In this perspective, currently ongoing initiatives, such as the ReportAGE-COVID of the Italian National Research Center on Aging (IRCCS INRCA) (ClinicalTrials.gov Id: NCT04348396) and the GeroCovid Initiative of the Italian Society of Gerontology and Geriatrics [58], as well as the Outcomes and prognostic factors in coronavirus disease (COVID-19) in very old intensive care patients (COVIP), a European multicenter study, (ClinicalTrials.gov Id: NCT04321265) aim at improving the knowledge of the disease and its interaction with frailty and multimorbidity in regards to health status and needs of care, as well as at promoting patient-centered health care strategies tailored to different settings and available resources [22].

## 5. Conclusions

Unarguably, early detection and careful monitoring of frailty represent a neglected strategy in the management of older adults with COVID-19. From the geriatricians’ point of view, the enormous cultural background on frailty built in decades of hard research work needs to be mandatorily transferred to real-world clinical practice. A frailty-based tailored management of the older population, involving primary care and geriatric services, may help to prevent an overwhelming demand for beds and hospital resources and the risk of another unacceptable hecatomb. Clinicians and researchers are called to collaborate in providing in-depth analysis from large-scale retrospective data to investigate the impact of frailty on COVID-19 interventions and to plan prospective studies able to bridge current knowledge gaps. Aware of the possibility of a second wave of COVID-19 or other pandemics, we have to prepare the boat to avoid being flooded again.

## Figures and Tables

**Table 1:** Summary of findings from retrieved studies.

Study	*N*	Age	Design and Setting	Outcome(s)	Frailty Assessment	Main Results
Baker, 2020 [47]	291	60–83	Retrospective Cohort Study Adults admitted to a large NHS Foundation Trust in UK with COVID-19	Hospital mortality	CFS	Patients who died without ventilatory support were generally frail with a median CFS of 7 (interquartile range = 6–7).
Bellelli, 2020 [42]	105	51.8–83.6	Retrospective observational study COVID-19 patients admitted to hospital	Hospital mortality or ICU admission	31-item Frailty Index	Frailty Index was independently associated with Hospital mortality or ICU admission (OR = 1.32, 95% CI = 1.03–1.70).
Brill, 2020 [43]	450	56–83	Retrospective cohort study Hospitalized patients with COVID-19	Hospital mortality *N* = 173 Hospital infection *N* = 31	CFS	Patients who died had greater median frailty (5, interquartile range = 3–6) compared to survivors (4, interquartile range = 3–5.5, *p* = 0.014). Such difference was more evident among patients aged 80 or more (6, interquartile range = 5–7 vs. 5, interquartile range = 4–6, *p* = 0.002). Patients who developed the infection during hospitalization were also more frail compared to those with community-acquired infection (5, interquartile range = 4–6 vs. 5, interquartile range = 3–6, *p* = 0·047)
Hoek, 2020 [46]	23	21–81	Retrospective cohort study Single organ transplant recipients with COVID-19	Mortality	CFS	Solid organ transplant recipients affected by COVID-19 who died had a mean CFS of 5.8 compared to 1.92 for survivors.
Miles, 2020 [44]	377	70–99	Prospective case-control study Hospitalized COVID-19 patients (217) vs. non-COVID-19 controls (*N* = 160)	All-cause mortality	CFS	For frailty, differences in effect size were evident between cases (HR = 1.02, 95% CI = 0.93–1.12) and non-COVID-19 controls (HR = 1.99, 95% CI = 1.46–2.72), with an interaction term (HR = 0.51, 95% CI 0.37–0.71) suggesting that frailty is not a good discriminator of prognosis in COVID-19.
Turner, 2020 [45]	36	48–96	Retrospective observational study Patients who died with COVID-19	Death phenotypes and disease trajectories	CFS	Higher frailty scores (CFS ≥ 5) and older age (>75 years) were found in the group (*n* = 14) characterized by longer duration of both disease and dying phases. In contrast, lower frailty scores were observed among patients with fulminant COVID-19 course and those characterized by long illness duration and stability phase followed by rapid death.

CFS, Clinical Frailty Scale; ICU, intensive care unit.
